# The Charcot–Marie–Tooth Neuropathy (CMTX3) Complex Structural Variation Causes Differential SOX3 Spatiotemporal Expression

**DOI:** 10.1155/humu/4225263

**Published:** 2026-09-11

**Authors:** Alexandra Boyling, Anthony N. Cutrupi, Desmond Li, Ben Crossett, Jonathan J. Danon, Edward D. Harvey-Latham, Garth A. Nicholson, Steve Vucic, Marina L. Kennerson

**Affiliations:** ^1^ Northcott Neuroscience Laboratory, ANZAC Research Institute, Sydney Local Health District, Sydney, New South Wales, Australia, anzac.edu.au; ^2^ School of Medical Sciences, Faculty of Medicine and Health, The University of Sydney, Sydney, New South Wales, Australia, sydney.edu.au; ^3^ Sydney Mass Spectrometry, The University of Sydney, Sydney, New South Wales, Australia, sydney.edu.au; ^4^ School of Chemistry, Faculty of Science, The University of Sydney, Sydney, New South Wales, Australia, sydney.edu.au; ^5^ Molecular Medicine Laboratory, Concord Hospital, Sydney, New South Wales, Australia, concordhospital.org; ^6^ Brain and Nerve Research Centre, Concord Clinical School, The University of Sydney, Sydney, New South Wales, Australia, sydney.edu.au

## Abstract

Charcot–Marie–Tooth (CMT) neuropathy is a clinically and genetically heterogeneous group of diseases characterized by the length‐dependent axonal degeneration of peripheral nerves. We previously mapped a rare form of X‐linked CMT, CMTX3, to a 5.7‐Mb interval on chromosome Xq26.3‐q27.1 and excluded the coding region of all known genes in the linkage interval for mutations. Whole genome sequencing subsequently identified a 78‐kb region of chromosome 8q24.3 that had been duplicated and inserted into the CMTX3 locus between the genes *HAPSTR2* and *SOX3*. The 78‐kb insertion, which contains a partial transcript of *ARHGAP39*, fully segregated in families with CMTX3 and was absent in neurologically normal controls. To retain the CMTX3 insertion and investigate its consequences in appropriate neuronal tissue, we generated induced pluripotent stem cells (iPSCs) from CMTX3 fibroblasts. Using bulk RNA sequencing of patient‐derived spinal motor neurons, *ARHGAP39* was deemed nonpathogenic by excluding both the formation of novel fusion transcripts and dosage effects from the partial duplication. Subsequent NanoString expression analyses of candidate genes within the CMTX3 locus, across different stages of neuronal differentiation, identified spatiotemporal dysregulation of *SOX3.* NanoString showed reduced *SOX3* expression in patient iPSCs. RNA sequencing detected *SOX3* downregulation in CMTX3 neuroepithelial progenitor cells, which was further confirmed by quantitative proteomics. Given the early onset and relatively rapid progression of CMTX3, these data prioritise *SOX3* as a leading candidate gene, consistent with its role as one of the earliest transcription factors expressed in the developing nervous system and a key regulator of neuronal fate.

## 1. Introduction

Charcot–Marie–Tooth (CMT) neuropathy is a clinically and genetically heterogenous disease, causing length‐dependent axonal degeneration of motor and sensory neurons. Patients present with progressive distal muscle weakness and atrophy, sensory impairment, and variable involvement of the central nervous system. The disease has a prevalence of 1 in 2500 [1]. CMTX3 is one of six X‐linked forms of CMT [2], with the most common being CMTX1, which is caused by disease‐associated variants in the gap junction beta 1 (*GJB1*) gene [3]. We mapped the CMTX3 locus in two distantly related families to a 5.7‐Mb interval on chromosome Xq26.3‐q27.3 [4]. After excluding all genes within the linkage locus for coding mutations, selected affected individuals underwent short‐read whole genome sequencing. A complex structural variation (SV) was subsequently identified in which a 78‐kb region of chromosome 8q24.3 was duplicated and inserted into the CMTX3 locus at a previously described Xq27.1 recombination “hotspot” between the genes *HAPSTR2* (formerly *LOC389895*) and *SOX3* [5]. Segregation analysis demonstrated the SV was present in all affected individuals (25 affected males and 30 carrier females) and absent in 50 unaffected family members as well as population controls [5]. This was the first report of a large insertion causing X‐linked CMT and highlighted the importance of expanding the spectrum of mutation screening to SV in cases where all coding mutations had been excluded in a family.

Identification of the CMTX3 complex SV has contributed to the growing evidence that large DNA rearrangements, particularly those located in noncoding or intergenic regions, may represent an underrecognized cause of inherited peripheral neuropathies. The change in the genomic landscape of the CMTX3 locus caused by the insertion introduced a duplicated portion of the *ARHGAP39* gene (NM_001308207.1; exons 1–7) from chromosome 8q24.3, with the closest genes to the 78‐kb insertion being *HAPSTR2* (formerly *LOC389895*) (located 329 kb proximal to insertion) and *SOX3* (located 84 kb distal to insertion). We proposed several hypotheses for the functional consequences of the CMTX3 insertion including gene dosage, the formation of novel fusion transcripts or alternative splicing, disrupting the interaction between a gene and its functional noncoding DNA sequences (such as promoters, enhancers, and silencers), or introducing new regulatory interactions, resulting in dysregulated temporal and spatial gene expression [5].

We have previously reviewed the impacts of SV in inherited peripheral neuropathies [6] and devised a strategy for investigating the pathogenic consequences of the CMTX3 SV, particularly at the Xq27.1 “hotspot,” where several other diseases have been reported due to the insertion of large, duplicated DNA fragments from different regions of the genome [7]. In the present study, we have used components of this strategy and generated patient‐derived iPSC lines from reprogrammed fibroblasts to retain the SV mutation and model CMTX3 at different stages of neuronal differentiation. We excluded the partial duplication of *ARHGAP39* as a causative mechanism and, using complementary transcriptomic approaches together with proteomics, identified *SOX3* as the lead candidate gene for CMTX3 based on its spatiotemporally specific downregulation in CMTX3‐derived tissue.

## 2. Materials and Methods

### 2.1. Ethics Statement

The protocols utilized in the current study (2019/ETH07839) have been approved by the Sydney Local Health District Human Ethics Review Committee. Informed consent was obtained from study participants.

### 2.2. Cell Line Summary

Although CMT is classified as a rare disorder, CMTX3 is ultrarare because of its exceptionally low prevalence [8], creating clear constraints on access to sufficient donor material for functional studies. Accordingly, the sample sizes in this study range from *n* = 1 to *n* = 3 CMTX3‐derived cell lines, reflecting the staged acquisition and expansion of donor material over the course of the project. Full details of the cell lines used in the present study are provided in Table S1.

### 2.3. Culturing and Maintenance of Primary Fibroblasts

Primary fibroblasts were maintained in F‐DMEM culture medium comprising DMEM, 10% (v/v) Foetal Bovine Serum, 1% (v/v) penicillin/streptomycin and 1% (v/v) L‐glutamine (Gibco, Thermo Fisher Scientific, Waltham, MA, USA). All cells were maintained in 5% CO_2_, humidified air at 37°C. Media was replaced every 2–3 days. Cells were maintained under these conditions for 7 days for biobanking or harvested (70%–80% confluency) for downstream experiments.

### 2.4. Generation, Culturing, and Maintenance of iPSC Lines

Reprogramming of patient and unrelated control fibroblasts was outsourced to the Stem Cell and Organoid Facility (Children′s Medical Research Institute, Westmead, NSW, Australia) and FUJIFILM Cellular Dynamics (Madison, WI, USA), where in‐house immunofluorescence was used to confirm the expression of multiple pluripotency markers. Reprogrammed iPSCs were shown to be karyotypically normal using either G‐banded karyotyping (WiCell; Madison, WI, USA) or digital SNP karyotyping with the Illumina Infinium GSA‐24 v3.0 microarray (Victorian Clinical Genetics Services, Melbourne, VIC, Australia). Description of reprograming methods used by FUJIFILM Cellular Dynamics has been detailed elsewhere [9]. Additional control lines were purchased from FUJIFILM Cellular Dynamics.

iPSCs were seeded in six‐well plates (Corning, New York, USA) coated with Matrigel (0.167 mg/mL, Corning) and maintained in TeSR‐E8 medium (STEMCELL Technologies, Vancouver, BC, Canada) that was replaced daily. Subculturing was performed using 0.5‐mM UltraPure EDTA (Invitrogen, Carlsbad, CA, USA) as described in Cutrupi et al. (2023) [9].

### 2.5. Differentiation of Neuronal Precursors and Mature Spinal Motor Neurons

iPSCs were differentiated into caudal neuroepithelial cells (NEP—Differentiation Day 6), motor neuron progenitors (MNP—Differentiation Day 12), neurospheres (NSPHR—Differentiation Day 18) and mature spinal motor neurons (sMNs—Differentiation Day 28) using previously described methods [9]. SN38‐P was used to further purify neuronal cultures from nonneuronal cells [10], as described in [9]. Adherent culturing was performed in six‐well plates (Corning‐Costar) pretreated with 0.167‐mg/mL Matrigel (Corning). Suspension culturing was performed in ultralow attachment six‐well plates (Corning‐Costar).

### 2.6. CMTX3 SV Genotyping PCR Assay

DNA was isolated from patient and control iPSCs using QuickExtract DNA Extraction Solution 1.0 (Epicentre, Madison, WI, USA) according to the manufacturer′s instructions. A multiplex PCR was used to genotype the CMTX3 complex insertion as previously described [5], with primers synthesized by Sigma‐Aldrich (St. Louis, MO, USA). Agarose gels (2% w/v) were prepared in 1 X TAE buffer (Astral Scientific) with 0.01% SYBR Safe DNA gel stain (Thermo Fisher Scientific). HyperLadder 100 bp (Bioline, London, UK) was used as a size standard. PCR products were size‐fractionated and visualized using a Safe Imager Transilluminator 2.0 (Invitrogen). Images were captured using a Canon PhotoShot S5‐IS digital camera with a Hoya O(G) filter.

### 2.7. Quantitative Real‐Time Polymerase Chain Reaction (qRT‐PCR) for the Confirmation of iPSC Pluripotency

Total RNA was isolated from CMTX3 (P1 and P2) and control (C1–C3) iPSCs as well as a single control fibroblast line (negative control) using the RNeasy Mini Kit (Qiagen, Hilden, Germany). A reverse‐transcribed template was prepared using the iScript cDNA Synthesis Kit (Bio‐Rad, Hercules, CA, USA). Quantitative RT‐PCR was performed using TaqMan Gene Expression Assays for six pluripotency markers (*LIN28A*, *NANOG*, *NODAL*, *FOXD3*, *CDH1*, and *TDGF1*) and one internal housekeeping gene (*GAPDH*) (Table S2). Reactions were prepared in 20‐*μ*L volumes containing 1X TaqMan Gene Expression Assay (Applied Biosystems, Foster City, CA, USA), 1X TaqMan Gene Expression Mastermix (Applied Biosystems), and 25‐ng cDNA template. Water was used as a negative control in all assays. Thermal cycling was performed (StepOnePlus Real‐Time PCR; Applied Biosystems) using the following cycling protocol: 95°C for 20 s, followed by 40 cycles of 95°C for 1 s and 60°C for 20 s. Data analysis was performed using the StepOne Plus Software (Version 2.1). GraphPad Prism (Version 10) was used to plot the raw *Ct* values, enabling a qualitative representation of the expression of pluripotency genes between iPSCs and fibroblasts [11, 12].

### 2.8. NanoString nCounter Targeted Gene Expression Analysis

Gene expression quantification using the NanoString nCounter gene expression system (NanoString Technologies, Seattle, WA, USA) was outsourced to Westmead Medical Research Institute (Sydney, NSW, Australia). A custom NanoString nCounter Elements TagSet panel was designed to detect 48 RNA targets, including 21 genes within and flanking the CMTX3 linkage region, one gene contained within the CMTX3 insertion sequence, 11 genes implicated in peripheral nervous system differentiation, and 14 housekeeping genes (Table S3). Housekeeping genes were selected in accordance with the NanoString technical guidelines, which recommend utilizing a combination of housekeeping genes with high, medium and low expression levels [13]. The target‐specific oligonucleotides were designed by NanoString Technologies (Seattle, WA, USA) and synthesized by Integrated DNA Technologies (Coralville, IA, USA). Total RNA was isolated from patient (*n* = 2) and control (*n* = 3) tissues using the RNeasy Mini Kit (Qiagen) and diluted to a concentration of 14.286 ng/*μ*L. RNA yield was assessed using NanoDrop (Thermo Fisher Scientific), and integrity was determined using a TapeStation (Agilent Technologies, Santa Clara, CA, USA). Details regarding assay preparation, execution and analysis are previously described [9]. For analyses involving multiple different cell types where housekeeping expression levels were quite variable, a selection of housekeeping genes showing minimal variability was utilized. The nSolver Analysis Software (NanoString Technologies) was used for statistical analysis of differential gene expression between patient and control samples. A Welch′s *t*‐test was performed using the log2‐transformed normalized count data as input and a significance threshold of *p* < 0.05.

### 2.9. RNA Sequencing (RNA‐Seq) Data Processing and Analysis

Total RNA was extracted from sMN cells (*n* = 1 patient (two clones), *n* = 3 controls) and NEP cells (*n* = 2 patients, *n* = 3 controls) using the RNeasy Mini Kit (Qiagen). Yields were quantitated using NanoDrop (Thermo Fisher Scientific), and the RNA integrity was determined using a 2200 TapeStation (Agilent Technologies). Poly(A) RNA‐seq, including library preparation and data processing, was outsourced to Macrogen (Seoul, South Korea) as previously described [9]. Briefly, novel fusion transcripts were predicted using three bioinformatic tools: deFuse (Version 0.8.1) [14], FusionCatcher (Version 1.00) [15], and Arriba (Version 1.2.0) [16]. To identify novel transcripts and splice variants, transcripts were assembled using StringTie (Version 2.1.3b) [17] and then classified with GffCompare [18]. Gene‐level differential gene expression analysis was performed using edgeR (Version 3.40.2) [19] and/or DESeq2 (Version 1.38.3) [20]. Differentially expressed genes (DEGs) were determined by log_2_FC ≠ 0 and a false discovery rate (FDR) adjusted *p* value threshold of < 0.05 (edgeR) or < 0.1 (DESeq2).

### 2.10. Immunofluorescence

Immunofluorescence experiments were performed as previously described [9]. In brief, cells were cultured in Nunc Lab‐Tek eight‐well chamber slides (Thermo Fisher Scientific) or black, optically clear PhenoPlate 96‐well microplates (Perkin Elmer, Waltham, MA, USA) coated in Matrigel (0.167 mg/mL; Corning). Cells were washed once in DPBS (Gibco), fixed in 4% (v/v) paraformaldehyde (PFA, Sigma‐Aldrich) for 20 min at room temperature, washed once with DPBS, treated with permeablization solution (DPBST) containing DPBS and Triton X‐100 (Calbiochem) ranging from 0.2% to 0.3% (v/v) (depending on density of cellular clumps) for 30 min at room temperature and blocked (DPBS and 5% (w/v) bovine serum albumin [Sigma Aldrich]) for 1 h at room temperature. Cells were incubated with primary antibodies overnight at 4°C. iPSCs: anti‐OCT4 (Cell Signaling Technology, Danvers, MA, USA, #2840, 1:400), anti‐SOX2 (Cell Signaling Technology, #3579, 1:400), and anti‐NANOG (Cell Signaling Technology, #4903, 1:400). MNP: anti‐OLIG2 (Millipore, Burlington, MA, USA, MABN50 1:100). sMN: anti‐MNX1 (Sigma‐Aldrich, HPA071717, 1:500), and anti‐TUBB3 (Sigma‐Aldrich, T2220, 1:1000). The cells were then incubated with Alexa Fluor (AF) secondary antibodies (Invitrogen) for 1 h at room temperature. The following secondary antibodies were used: goat anti‐rabbit AF 488 (Invitrogen, A11008, 1:500), goat anti‐rabbit AF 555 (Invitrogen, A21428, 1:500), goat anti‐mouse AF 488 (Invitrogen, A11001, 1:500), and goat anti‐mouse AF 555 (Invitrogen, A21422, 1:500). Nuclei were counterstained with 4,6‐diamidino‐2‐phenylindole (DAPI, Molecular Probes, 0.2 *μ*g/mL) and mounted with mounting media (75% glycerol (v/v) and 20‐mM Tris, pH 8). Samples were imaged on a Leica SP8 confocal microscope (Leica Microsystems, Wetzlar, Germany) and visualized using Leica Application Suite X (LAS X) software (Leica Microsystems). Images were processed using FIJI [21] (Version 2.14.0) for Windows.

### 2.11. Quantification of MNX1‐Positive sMN

FIJI (Version 2.14.0) was used to generate maximum intensity Z‐projection images for downstream analysis. Images of sMN were obtained from three separate rounds of differentiation (*n* = 2 patients and *n* = 3 controls), with cells stained 8 days after neuron plating. A custom CellProfiler [22] (Version 4.2.5) pipeline was used to calculate the percentage of sMN nuclei positive for MNX1. First, the “IdentifyPrimaryObjects” module was used to identify DAPI‐stained nuclei. A global minimum cross‐entropy threshold method was employed. To determine the lower threshold limit, images were manually inspected using the “pixel intensity” tool to provide intensity values for background fluorescence. To determine the nuclei diameter range, the “measure length” tool was used to manually assess the typical diameter of nuclei within the fluorescence images. Optimal segmentation of nuclei was achieved by declumping objects based upon intensity. However, the presence of small, bright DAPI speckles introduced the risk of false‐positive nuclei identification, which could have significant effects on downstream analysis. To overcome this issue, the “FindMaxima” module was used to identify high‐intensity DAPI speckles, which were then processed using the “ConvertImageToObjects” module and subsequently excluded from the image using the “MaskObjects” function. Masking settings were optimized to retain partially masked objects if they display high overlap with the masking region. This setting minimizes the risk of nuclei being discarded in cases where they colocalize with the DAPI speckles. To identify the number of MNX1‐positive cells, the MNX1‐stained images were processed using the “IdentifyPrimaryObjects” module, as described above. The “RelateObjects” module was used to associate the two sets of identified objects (i.e., the DAPI objects and the MNX1 objects) with each other. Nuclei were then filtered based on the presence of MNX1‐staining using the “FilterObjects” module. The number of MNX1‐positive nuclei was divided by the total number of nuclei to calculate the proportion of sMN showing positive expression of MNX1. Proportion data underwent arcsine square root transformation prior to statistical testing. Statistical analysis was performed in GraphPad Prism (Version 10) using an unpaired two‐tailed *t*‐test with a significance threshold of *p* < 0.05.

### 2.12. Quantification of SOX3 by Tandem LC‐MS/MS

Cell pellets were resuspended in lysis buffer (4% sodium deoxycholate, 100‐mM Tris, pH 8.5) and heated at 95°C for 10 min. Pellets were lysed by sonication using a Branson Digital Sonifier SFX 150 (Emerson, MO, USA) in two rounds at 30% power for 30 s each. Debris was pelleted by centrifugation at 16,000 rcf for 10 min, and supernatant was collected. Cysteines were reduced and alkylated by incubation with 10‐mM Tris(2‐carboxyethyl)phosphine (TCEP) and 40‐mM chloroacetamide at 95°C for 10 min. Samples were diluted 4‐fold with water and digested with sequencing‐grade modified porcine trypsin added at a 1:20 ratio (trypsin:protein content) for 16 h at 37°C. Digested samples were cleaned by SDB‐RPS stage tips (styrenedivinylbenzene—reverse phase sulfonate) as per [23]. Briefly, samples were mixed with an equal volume of 99% ethyl acetate, 1% TFA to extract the SDC and centrifuged. The lower phase was cleaned by binding to an SDB‐RPS stage tip and washed with 99% ethyl acetate, 1% TFA, and then 0.2% TFA and eluted using 5% ammonium hydroxide and 80% acetonitrile and dried. Samples were reconstituted in 50 mM TEAB (triethylammonium bicarbonate), pH 8.5 and 20 *μ*g of peptide from each sample was labelled with TMT6plex isobaric labelling reagent (Thermo Fisher Scientific), following the manufacturer′s instructions. The TMT labelled peptides were combined and fractionated by high pH reverse‐phase chromatography using a 4.6 mm × 250 mm C18 column (3.5‐*μ*m particle size, Xbridge Premier BEH C18; Waters, MA, USA) on a Vanquish fraction collector (Thermo Fisher Scientific) with mobile phases A (10‐mM ammonium formate, 2% v/v acetonitrile, pH 8.5) and B (10‐mM ammonium formate, 80% v/v acetonitrile). Peptides were eluted using a linear gradient of 10% to 40% B over 11 mins at a flow rate of 1 mL/min. Sixteen concatenated fractions were collected and dried down prior to LC‐MS analysis.

Fractions were reconstituted in 3% acetonitrile v/v, 0.1% formic acid v/v and separated on an in‐house packed column (75 *μ*m × 500 mm, 1.9‐*μ*m ReproSil Pur 120 C18, Dr. Maisch GmbH, Germany) with a Vanquish Neo LC (Thermo Fisher Scientific) with mobile phase A (0.1% v/v formic acid) and B (80% v/v ACN, 0.1% FA v/v). Peptides were eluted at a flow rate of 0.3 *μ*L/min and a gradient of 3% B to 40% B in 66 min, 40% B to 60% B in 2 min, and 60% B to 98% B in 2 min and then washed with 98% B while increasing flow from 0.3 to 0.5 *μ*L/min over 5 min. The column was then equilibrated at 3% B for 2.7 column volumes at 750 bar. The LC was coupled to an Orbitrap Eclipse mass spectrometer (Thermo Fisher Scientific) with a spray voltage of 2300 V, RF lens of 30%, and ion transfer tube heated to 300°C. The Orbitrap Eclipse was operated in data‐dependent acquisition (DDA) mode comprising a survey MS scan acquired as a profile with a maximum injection time of 118 ms and a standard AGC target across a scan range from 350 to 1400 m/z with Orbitrap resolution of 60,000 with a 1.5‐s cycle time. MS/MS scans were performed between MS scans isolating the most abundant ions (1.4‐m/z isolation window, > 5e4 ions) with charge states +2 to +4 and a dynamic exclusion of 30 s. MS/MS scans were acquired with a maximum injection time of 54 ms and a normalized AGC target of 400% with a scan range starting from 100 m/z, HCD collision energy of 35% and Orbitrap resolution of 30,000.

The data were analysed using Proteome Discoverer vr 2.5 (Thermo Fisher Scientific) and Mascot vr 3.1 [24] (Matrix Science, London, UK) using the machine learning model MS2PIP:HCD2019 [25]. The search parameters included the following variable modifications: acetylation (protein N‐terminal), oxidation (M), deamination (NQ), carbamidomethyl (C), TMT6plex (K‐ and N‐term), and trypsin enzyme allowing for two missed cleavages and 10‐ppm precursor and fragment mass tolerance. The Swiss‐Prot Homo Sapiens database (UP000005640) was used, and results were filtered at 1% FDR. The TMT reporter fragments were used for quantification. Differential protein expression was performed in RStudio using the Differential Expression analysis of Proteomics data (DEP) tool [26].

## 3. Results

### 3.1. Characterization of CMTX3 Patient‐Derived iPSC Lines

**Figure 1:**
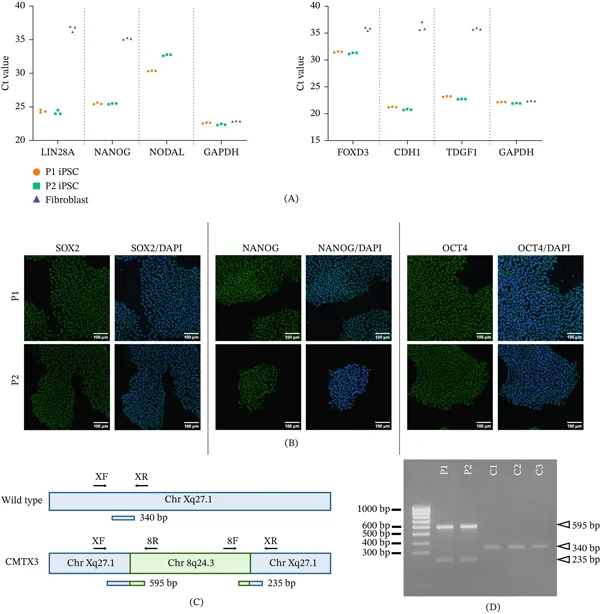
Characterization and genotyping of CMTX3 patient‐derived iPSC. (A) RT‐qPCR assessment of pluripotency‐associated transcripts (*LIN28A*, *NANOG*, *NODAL*, *FOXD3*, *CDH1*, and *TDGF1*) in two independent CMTX3 iPSC lines (P1 and P2) compared with fibroblasts. *GAPDH* was used as an endogenous control. As *NODAL* was undetectable in fibroblasts, data are represented as raw *Ct* values. (B) Immunofluorescence staining confirming pluripotency marker expression (SOX2, NANOG, and OCT4; green) in CMTX3 iPSC (P1 and P2). Nuclei are counterstained with DAPI (blue). Scale bars, 100 *μ*m. (C) Schematic of the CMTX3 structural variant and genotyping primer (F = forward and R = reverse) locations indicating the expected amplicons from the wild‐type X chromosome (340 bp) and CMTX3 junction fragments (595 and 235 bp) (adapted from Brewer et al. [5], created with BioRender). (D) Genotyping PCR and gel electrophoresis confirms the presence of the CMTX3 SV in patient‐derived iPSC lines (P1 and P2) and their absence in control lines (C1‐C3). HyperLadder 100 bp (Bioline) was used as a size standard. Agarose gel (2% w/v) was prepared in 1X TAE buffer containing 0.01% SYBR Safe (Thermo Fisher Scientific).

Reprogramming of CMTX3 patient fibroblasts into iPSCs was outsourced to FUJIFILM Cellular Dynamics (Madison, WI, USA) and the Stem Cell and Organoid Facility (Children′s Medical Research Institute, Westmead, NSW, Australia), where pluripotency was initially validated using established in‐house protocols. Following receipt of the patient‐derived iPSCs, pluripotency was independently reconfirmed at both the transcript and protein levels by qPCR and immunofluorescence staining, respectively (Figure 1A,B). To verify that the disease‐causing CMTX3 SV was retained throughout the reprogramming process, patient and control iPSC lines were genotyped using a previously established multiplex PCR assay designed to detect the CMTX3 SV breakpoint junctions [5]. Figure 1C,D shows that the breakpoint junction fragments were successfully amplified in CMTX3 iPSC lines but absent in control lines, confirming preservation of the pathogenic SV following reprogramming.

### 3.2. Generation of MNP and sMN by Differentiation of CMTX3 Patient‐Derived iPSC Lines

**Figure 2:**
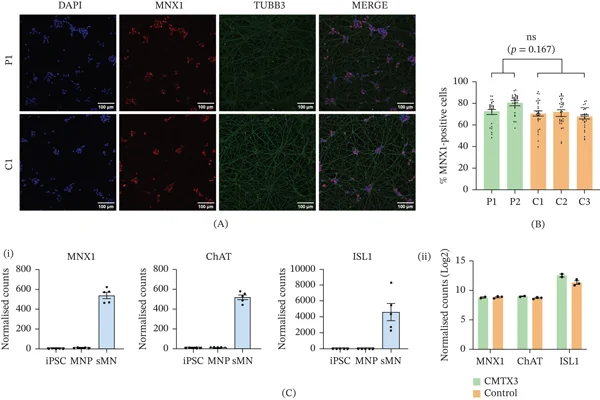
iPSC‐derived sMNs express canonical motor neuron markers. (A) Representative immunofluorescence images of terminally differentiated sMNs from a CMTX3 patient (P1) and control (C1) showing MNX1 (motor neuron identity; red) and *β*III‐tubulin/TUBB3 (neuronal cytoskeleton; green). Nuclei are counterstained with DAPI (blue); merged images are shown. Scale bars, 100 *μ*m. (B) Quantification of MNX1‐positive nuclei across CMTX3 patient (P1 and P2) and control (C1–C3) lines. Points represent the proportion of MNX1‐positive nuclei from acquired images collected over three independent differentiations. Error bars represent mean ± SEM. No significant difference in MNX1+ yield was detected between CMTX3 and controls (unpaired two‐tailed Student′s *t*‐test; ns *p* = 0.167). (C) NanoString nCounter analysis of motor neuron marker expression. Error bars represent mean ± SEM. (i) Normalized mRNA counts for *MNX1*, *ChAT*, and *ISL1* are shown at various differentiation stages (iPSC, MNP, and sMN; *n* = 5 cell lines per stage). (ii) Log2‐transformed normalized counts for the same markers in sMN, comparing CMTX3 patient (*n* = 2) versus control (*n* = 3) samples. Statistical analysis for each marker conducted using an unpaired *t*‐test with Welch′s correction and a significance threshold of *p* < 0.05. No significant differences were detected for MNX1 (*p* = 0.756), ChAT (*p* = 0.110), or ISL1 (*p* = 0.056).

To generate a motor neuron model for CMTX3, two patient‐derived and three control iPSC lines were differentiated into sMN by highly expandable OLIG2‐positive motor neuron progenitors (MNP) as previously described [9]. Efficient ventralization was confirmed by immunofluorescence, which demonstrated robust OLIG2 expression at the MNP stage (Figure S1A). Consistent with this, NanoString nCounter profiling consistently showed marked upregulation of *OLIG2* in MNPs compared with iPSCs, where expression was undetectable (Figure S1B), confirming successful generation of MNPs. Terminal differentiation of MNPs to mature sMNs was subsequently performed under 3D culture conditions as previously described [9]. Immunofluorescence analysis showed robust expression of the motor neuron marker MNX1 and the neuronal cytoskeletal marker *β*III‐Tubulin (TUBB3) in mature sMNs (Figure 2A). Quantification revealed no significant difference in the proportion of MNX1‐positive sMNs between CMTX3 cultures (76.53% ±3.966 SEM) and controls (69.99% ±1.418 SEM) (Figure 2B; *p* = 0.167). Similarly, NanoString analysis demonstrated increased expression of motor neuron markers *MNX1*, *ISL1*, and *ChAT* during differentiation, with the highest expression observed in sMNs relative to iPSCs and MNPs (Figure 2C [i]). No significant differences in expression of these markers were detected between CMTX3 and control sMN cultures (Figure 2C [ii]) indicating comparable differentiation efficiency and motor neuron identity across lines.

### 3.3. Exploratory RNA‐Seq of Patient‐Derived sMN Reveals No Novel Fusion Transcripts or Aberrant Splicing Associated With the CMTX3 SV

Two leading hypotheses have been proposed to explain the pathomechanism underlying CMTX3. The first implicates the partial *ARHGAP39* sequence contained within the CMTX3 insertion, whereby disease may result from overexpression of the duplicated sequence, production of a truncated *ARGHAP39* transcript or the formation of a novel fusion transcript species [5]. The second hypothesis suggests the ~78‐kb SV perturbs the local regulatory landscape at Xq27.1, leading to dysregulation of one or more neighboring genes [5]. To investigate both possibilities, bulk RNA‐seq was performed on patient‐derived sMNs, providing an unbiased assessment of transcriptional consequences of the CMTX3 SV. Analyses were conducted using sMNs from one affected individual represented by two iPSC clones and compared with three control lines. Quality control metrics of all samples are summarized in Table S4.

None of the three fusion‐calling algorithms identified fusion transcripts with breakpoints spanning the CMTX3 linkage region on chromosome X and the duplicated 8q24.3 sequence, providing no evidence that the inserted DNA generates a pathogenic fusion transcript (Table S5). Among fusion transcripts with both breakpoints on chromosome X, none mapped to the CMTX3 linkage interval (Tables S6, S7, and S8). Although deFuse predicted several *ARHGAP39*‐associated fusion events, these were considered unlikely pathogenic due to (a) one of the fusion breakpoints being located outside the duplicated 8q24.3 sequence contained within the CMTX3 insertion, and (b) identical fusion transcripts were also detected in all three control lines, indicating they represent background transcriptional events rather than disease‐specific consequences of the CMTX3 SV (Table S9). Consistent with these findings, no additional fusion transcripts involving the duplicated fragment of 8q24.3 were identified by any of the fusion‐calling tools (Tables S10 and S11). Collectively, these results provide no evidence that CMTX3 pathogenesis is mediated through the generation of a novel fusion transcript.

Analysis of novel transcript formation identified no transcripts incorporating sequence from either the duplicated 8q24.3 insertion fragment (Table S12A) or the broader CMTX3 locus at Xq27.1 (Table S12B). No novel splice variants originated from within the 8q24.3 insertion sequence itself (Table S13A). Within the CMTX3 linkage interval, eight novel splice variants were detected in both patient and control samples and therefore lacked disease specificity (Table S13B).

To investigate whether the CMTX3 SV results in local gene dysregulation, differential gene expression analysis was performed on patient‐derived and control SMNs. As biological replicates from independent CMTX3 patients were not available in this preliminary experiment, the analysis was considered exploratory and was aimed at identifying candidate genes for subsequent validation. Differential expression was assessed using edgeR, a statistical framework well suited to RNA‐seq datasets with limited sample numbers [19]. A total of 52 DEGs were identified; however, none mapped to either the CMTX3 linkage interval at Xq27.1 or to the duplicated ~78‐kb fragment derived from 8q24.3 (Table S14). To specifically evaluate local transcriptional effects, positional candidate genes within the CMTX3 locus were evaluated separately. Table 1 summarizes log2(fold change) values for 29 genes located within or adjacent to the affected genomic region. Although none reached statistical significance following multiple‐testing correction (FDR > 0.05), this analysis provided an overview of the local transcriptional landscape in patient and control sMNs. Of the 29 positional candidates, 17 were excluded from differential expression testing because of low expression levels in sMNs. Among the remaining genes, most exhibited modest changes with average absolute log2(fold change) values below 0.5, with the exception of two genes, *SOX3* and *ZIC3* (Table 1). However, *ZIC3* expression was highly variable between the two patient‐derived clones (Clone A = −2.185 and Clone B = −0.094) reducing confidence in its relevance. In contrast, *SOX3* showed a consistent trend of downregulation across both patient clones (Clone A logFC −1.383 and Clone B logFC −1.341); however, its overall expression levels were low (mean TPM 0.567 in the patient clones and 1.477 TPM in controls).

**Table 1:** Exploratory differential expression of positional candidate genes in patient‐derived sMN. Positional candidates include all RefSeq‐curated genes located within the CMTX3 linkage interval at Xq27.1 together with the partially duplicated *ARHGAP39* gene contained within the inserted 8q24.3 fragment. RNA‐seq was performed on sMN mRNA isolated from a single CMTX3 patient (P1) represented by two independent iPSC clones (P1 Clone A and Clone B) and 3 unrelated control lines. Differential expression was analysed using edgeR, with each patient clone independently compared against the control cohort (i.e., one vs. three). LogFC values represent gene expression changes in patient samples relative to controls. LogFC = log2(fold change); TPM = transcripts per kilobase million; FDR = false discovery rate. Genes designated NA were excluded from differential expression analysis due to low/undetectable counts.

Genes	CMTX3 P1 (clone A)				CMTX3 P1 (clone B)				Average LogFC
	LogFC	TPM	*p* value	FDR	LogFC	TPM	*p* value	FDR	
SOX3	−1.383	0.555	0.021	0.580	−1.341	0.580	0.025	0.696	−1.362
ZIC3	−2.185	0.199	0.019	0.557	−0.094	0.904	0.974	1.000	−1.139
FGF13‐AS1	−0.406	0.190	0.920	1.000	−0.517	0.182	0.881	1.000	−0.462
LDOC1	−0.147	57.074	0.341	1.000	−0.235	54.791	0.134	1.000	−0.191
LINC00632	−0.045	21.621	0.964	1.000	−0.275	18.838	0.590	1.000	−0.160
ATP11C	−0.135	26.080	0.435	1.000	−0.140	26.060	0.432	1.000	−0.138
ARHGAP39	−0.062	33.932	0.732	1.000	−0.191	31.664	0.292	1.000	−0.126
FGF13	0.032	28.670	0.821	1.000	−0.210	25.448	0.176	1.000	−0.089
MCF2	−0.161	9.560	0.560	1.000	0.128	12.045	0.593	1.000	−0.016
MAGEC3	0.029	0.277	0.975	1.000	0.026	0.436	0.951	1.000	0.028
LOC389895^a^	0.114	6.007	0.754	1.000	0.063	5.930	0.854	1.000	0.088
SPANXA2‐OT1	−0.079	0.126	1.000	1.000	0.988	0.290	0.288	1.000	0.455
CDR1	NA	0.066	NA	NA	NA	0.160	NA	NA	NA
CXorf66	NA	0.000	NA	NA	NA	0.000	NA	NA	NA
F9	NA	0.000	NA	NA	NA	0.000	NA	NA	NA
LOC645188	NA	0.000	NA	NA	NA	0.000	NA	NA	NA
LOC728660	NA	0.000	NA	NA	NA	0.000	NA	NA	NA
MAGEC1	NA	0.000	NA	NA	NA	0.000	NA	NA	NA
MAGEC2	NA	0.000	NA	NA	NA	0.000	NA	NA	NA
MIR320D2	NA	0.000	NA	NA	NA	0.000	NA	NA	NA
MIR504	NA	0.000	NA	NA	NA	0.000	NA	NA	NA
MIR505	NA	0.000	NA	NA	NA	0.000	NA	NA	NA
SPANXA1	NA	0.000	NA	NA	NA	0.000	NA	NA	NA
SPANXA2	NA	0.000	NA	NA	NA	0.000	NA	NA	NA
SPANXB1	NA	0.000	NA	NA	NA	0.000	NA	NA	NA
SPANXC	NA	0.000	NA	NA	NA	0.000	NA	NA	NA
SPANXD	NA	0.000	NA	NA	NA	0.000	NA	NA	NA
SPANXN4	NA	0.000	NA	NA	NA	0.000	NA	NA	NA
SRD5A1P1	NA	0.005	NA	NA	NA	0.065	NA	NA	NA

^a^ *LOC389895* is now termed *HAPSTR2.*

### 3.4. Overexpression of the Partially Duplicated *ARHGAP39* Is Not Detected in CMTX3 Patients

**Figure 3:**
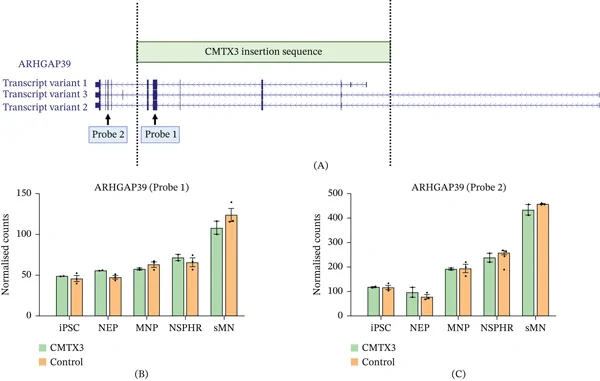
*ARHGAP39* expression across iPSC to sMN differentiation. (A) Schematic of *ARHGAP39* RefSeq curated transcript structure (UCSC Genome Browser, GRCh38) and the position of NanoString nCounter probes relative to the CMTX3 insertion sequence (green box). Probe 1 targets an exon within the partially duplicated *ARHGAP39* segment contained within the CMTX3 insertion. Probe 2 targets an exon outside the duplicated region. Created with BioRender. (B, C) Normalized NanoString nCounter counts for *ARHGAP39* measured using Probe 1 (B) and Probe 2 (C) in CMTX3 patient‐derived (*n* = 2) and control (*n* = 3) lines across five stages of sMN differentiation. Data are shown as mean ± SEM. To enable visualization across multiple cell types, the three housekeeping genes showing the lowest %CV were utilized for normalization and analysis in this figure. Statistical analysis for each timepoint was conducted on Log2‐transformed normalized counts using an unpaired *t*‐test with Welch′s correction and a significance threshold of *p* < 0.05. No significant differences were detected at any timepoint for either probe. Probe 1: iPSC, *p* = 0.479; NEP, *p* = 0.074; MNP, *p* = 0.240, NSPHR, *p* = 0.433, sMN, *p* = 0.283. Probe 2: iPSC, *p* = 0.836; NEP, *p* = 0.512; MNP, *p* = 1.00; NSPHR, *p* = 0.460; sMN, *p* = 0.479. iPSC = induced pluripotent stem cell, MNP = motor neuron progenitor, NEP = caudal neuroepithelial progenitor, NSPHR = neurosphere, and sMN = spinal motor neuron.

To further assess whether the partial duplication of *ARHGAP39* results in transcript overexpression in CMTX3, NanoString nCounter analysis was performed using two independent probes. One targeted an exon localized within the duplicated sequence of the CMTX3 SV and the second targeted an exon outside the duplicated region. Across all stages of differentiation analysed from iPSCs to mature sMN, both probes demonstrated comparable expression levels between patient and control samples (Figure 3).

### 3.5. *SOX3* Is Spatiotemporally Dysregulated in CMTX3 sMN Differentiation

**Figure 4:**
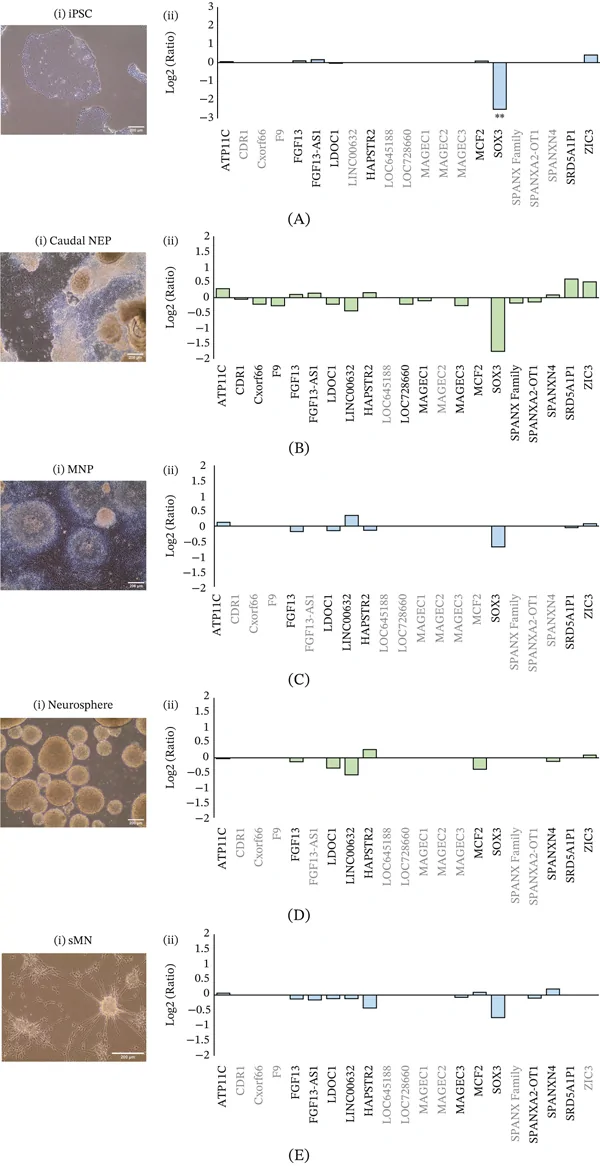
Relative expression of positional candidate genes throughout various stages of iPSC‐to‐sMN differentiation. RNA was isolated from CMTX3 (*n* = 2) and control (*n* = 3) samples at five discrete timepoints throughout the sMN differentiation process. (A) iPSC, (B) caudal NEP (Differentiation Day 6), (C) MNP (Differentiation Day 12), (D) neurospheres (Differentiation Day 18), and (E) sMN (Differentiation Day 28). Candidate gene expression was analysed using a custom NanoString nCounter panel. (i) Brightfield microscope images at each timepoint (scale bar = 200 *μ*m). (ii) Relative expression of positional candidate genes, expressed as the log2(Ratio) of CMTX3 versus control. Candidate genes with undetectable expression levels in all samples are denoted in gray font. Statistical analysis was performed using nSolver Analysis software (unpaired Welch′s *t*‐test on Log2‐transformed normalized count data). ∗∗ *p* <0.01.

**Figure 5:**
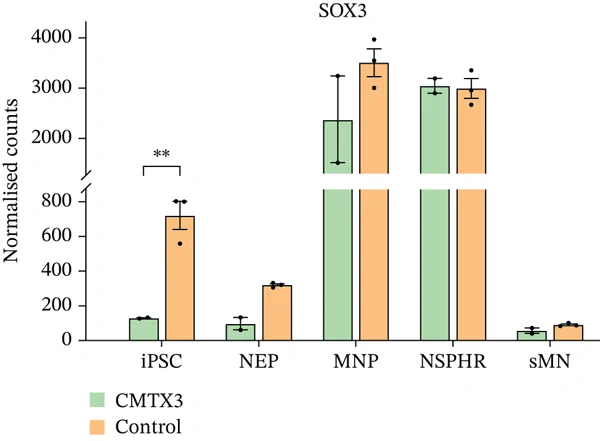
*SOX3* expression during iPSC‐to‐sMN differentiation in CMTX3. Nanostring nCounter counts for *SOX3* in CMTX3 patient‐derived lines (*n* = 2) and controls (*n* = 3) across five stages of differentiation. To enable visualization across multiple cell types, the three housekeeping genes showing the lowest %CV were utilized for normalization and analysis in this figure. Statistical analysis for each timepoint was conducted on Log2‐transformed normalized counts using an unpaired *t*‐test with Welch′s correction and a significance threshold of *p* < 0.05. *SOX3* was reduced in CMTX3‐derived iPSC relative to controls (*p* = 0.004), whereas no significant differences in expression were detected at other timepoints tested (NEP, *p* = 0.186; MNP, *p* = 0.439; NSPHR, *p* = 0.821; sMN, *p* = 0.317). Error bars show mean ± SEM. ∗∗ *p* <0.01.

In parallel with the global transcriptomic analysis by RNA‐seq, the NanoString nCounter platform was used to conduct a targeted assessment of local gene regulation within the CMTX3 linkage interval. Custom probes were designed to measure the expression of 21 positional candidate genes located within ~3 Mb on either side of the CMTX3 SV (Table S3). Gene expression was profiled across five stages of sMN differentiation (Figure 4A–E). At the iPSC stage, 12 of 21 candidate genes showed no detectable expression. Among the remaining genes, *SOX3* was the only transcript significantly differentially expressed between CMTX3 and control iPSC lines (*p* = 0.0012), exhibiting an approximately 5.7‐fold reduction in patient‐derived iPSCs (log2(Ratio) = ‐2.51) (Figure 4A). At the caudal NEP stage, no candidate gene reached statistical significance; however, *SOX3* again demonstrated a marked trend towards reduced expression in CMTX3 samples (log2(Ratio) = −1.75, *p* = 0.157) (Figure 4B). No significant differences in the expression of positional candidate genes, including *SOX3*, were detected at subsequent stages of differentiation (MNP, NSPHR, and mature sMN) (Figure 4C‐E). Together, these results identify a spatiotemporally restricted reduction in *SOX3* expression that is most prominent at the iPSC stage. This pattern prioritizes *SOX3* as a leading candidate for functional investigation in CMTX3. The temporal dynamics of *SOX3* expression throughout sMN differentiation are shown (Figure 5).

Given the established role of SOX3 as a transcription factor regulating early nervous system development [27], RNA‐seq was performed on caudal NEP cultures from patients (*n* = 2) and controls (*n* = 3) to further investigate the downstream transcriptomic consequences of CMTX3‐associated *SOX3* dysregulation. Quality control metrics are available in Table S15. Differential gene expression analyses using DESeq2 [20] and edgeR [19] identified 14 and 16 DEGs, respectively. Notably, *SOX3* was one of the 14 DEGs detected by DESeq2, and was the only DEG located within the CMTX3 linkage region (Table 2A). CMTX3 NEPs exhibited a ~3.89‐fold (log2(FC) = −1.96) reduction in *SOX3* expression relative to controls (adj. *p* = 0.0015), consistent with the trend observed in the NanoString nCounter analysis. In contrast, none of the 16 DEGs detected by edgeR mapped to chromosome X (Table 2B). Although *SOX3* did not formally reach statistical significance, it only narrowly missed the threshold for DEG classification by edgeR (log2(FC) = −1.966, FDR = 0.0506). To exclude alternative pathogenic mechanisms, NEP RNA‐seq datasets were also interrogated for novel fusion transcripts, aberrant splicing events or novel transcript species involving the CMTX3 insertion or surrounding genomic regions, using the same analytical framework applied to the sMNs. No CMTX3‐specific fusion, splicing, or transcript abnormalities were detected that involve the sequence of interest.

**Table 2:** Differentially expressed genes in caudal neuroepithelial progenitors from CMTX3 versus control lines. RNA‐seq was performed on poly(A) selected RNA isolated from caudal NEPs (Differentiation Day 6) derived from CMTX3 (*n* = 2) and control (*n* = 3) lines. (A) Differential gene expression analysis using DESeq2. Log2FoldChange values were calculated relative to the “control” group, and genes with an adjusted *p* value of < 0.1 (default threshold) were considered significantly differentially expressed. Genes are ranked according to adjusted *p* value. (B) Differential gene expression using edgeR. Log2FoldChange values were calculated relative to the control reference group, and significance was defined as false discovery rate (FDR) < 0.05. Genes are ranked according to FDR.

(a)			
Gene	Log2FoldChange	Adjusted *p* value	Chr
LINC01139	3.671	1.30861E‐11	1
EIF2S3B	3.781	1.58463E‐06	12
TMX2‐CTNND1	3.398	9.82779E‐06	11
PLOD2	−1.151	1.25649E‐05	3
SOX3	−1.962	0.00149	X
REC8	−1.948	0.00303	14
TRNY	−1.127	0.00367	MT
AHRR	−9.207	0.01918	5
TMEM132C	−1.343	0.01954	12
PLEKHA2	−1.277	0.02150	8
TRNS1	−1.137	0.03343	MT
LOC112268219	−7.415	0.03343	1
MIR600HG	−4.269	0.03622	9
CASKIN1	−0.910	0.06108	16
(b)			
**Gene**	**Log2FoldChange**	**FDR**	**Chr**
LINC01139	3.660	2.33E‐06	1
TEKT4P2	11.275	1.33E‐05	21
EIF2S3B	3.765	5.94E‐05	12
HLA‐DRB1	7.946	9.09E‐05	6
TMX2‐CTNND1	3.392	0.00043	11
LOC112268219	−7.960	0.00064	1
PLOD2	−1.156	0.00328	3
BORCS7‐ASMT	−7.637	0.00594	10
REC8	−1.954	0.00594	14
TMX2P1	6.849	0.01027	9
MRPL23‐AS1	−7.212	0.01368	11
CCDC38	6.805	0.01416	12
TMEM132C	−1.348	0.02414	12
TRNY	−1.132	0.02414	MT
TDGF1	6.514	0.03495	3
RPL10P9	6.588	0.03839	5

### 3.6. Proteomics Confirms Downregulation of SOX3 in CMTX3 Neuroepithelial Cells

**Figure 6:**
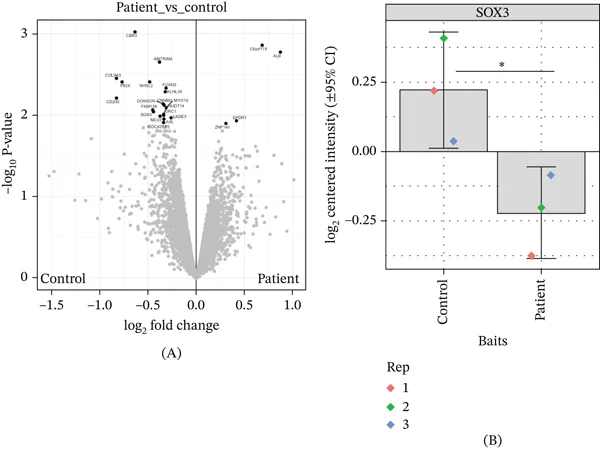
Proteomic profiling identifies reduced SOX3 abundance in CMTX3 patient‐derived samples. (A) Volcano plot showing differential protein abundance between CMTX3 patient (*n* = 3) and control (*n* = 3) samples. The *x*‐axis indicates log2 fold change (patient vs. control), and the *y*‐axis shows −log10 *p* value. Proteins meeting the significance threshold are highlighted (black dots), and selected proteins are annotated. (B) Quantification of SOX3 protein abundance in control and CMTX3 patient samples, plotted as log2‐centered intensity with 95% confidence intervals. Colored points denote individual biological replicates (Rep 1–3). Differential expression was determined using DEP^26^. The asterisk (∗) denotes a significant difference between groups (adj. *p* < 0.05).

To validate the temporal dysregulation of *SOX3* observed at the transcript level, iPSCs from CMTX3 patients (*n* = 3) and controls (*n* = 3) were differentiated into caudal NEP and harvested for TMT LC‐MS/MS proteomic analysis. Following TMT fragment quantification, removal of duplicate features and filtering to retain proteins identified in all samples from at least one condition, 8000 proteins were quantified per sample (Figure S2). Only three proteins were encoded by genes residing within the CMTX3 linkage interval (ATP11C, SOX3, ARHGAP39; Figure S3). Proteome‐wide differential abundance analysis identified 23 proteins that were significantly altered between CMTX3 and control NEPs (adj. *p* < 0.05, Figure 6A and Table S16). Notably, SOX3 was the only differentially abundant protein encoded within the CMTX3 linkage region (adj. *p* = 0.0213). Consistent with the transcriptomic analyses, SOX3 protein abundance was significantly reduced in CMTX3 NEPs relative to controls (Log2FC = −0.442, Figure 6B).

## 4. Discussion

Detecting complex pathogenic mutations has been greatly enhanced by modern advances in genomic sequencing technologies. The challenge now lies in interpreting their functional consequences. This is exemplified by CMTX3, in which the causative SV lies in noncoding DNA and does not disrupt any of the > 130 genes [28] known to cause CMT or related neuropathies. Establishing a mechanistic link between the CMTX3‐associated genotype and clinical phenotype has been challenging.

In this study, we generated the first neuronal model of CMTX3, enabling the opportunity to investigate pathomechanisms in relevant cell types that retain the disease‐associated SV. This is critical because the two leading hypotheses for CMTX3 pathogenesis (local gene dysregulation and generation of a novel transcript) are transcriptional mechanisms likely to be highly dependent on cellular and developmental context. We therefore evaluated both hypotheses in parallel using two independent and complementary transcriptomic platforms: short read RNA‐seq and NanoString nCounter analysis.

Across all stages assessed during iPSC to sMN differentiation, neither RNA‐seq nor NanoString nCounter analysis provided evidence of aberrant expression of the partially duplicated *ARHGAP39* region within the CMTX3 locus. RNA‐seq identified no CMTX3‐specific fusion transcripts, novel transcript isoforms, or aberrant splicing variants involving *ARHGAP39* in either NEPs or sMNs. Consistent with this finding, NanoString nCounter analysis showed comparable expression of an exon located within the duplicated fragment and an exon outside the duplicated region. Together, these results provide strong evidence against *ARHGAP39* as the gene underlying CMTX3. In contrast, both transcriptomic platforms independently demonstrated reduced expression of the positional candidate, *SOX3*, in patient‐derived cells. This reduction was confined to early developmental stages (iPSCs and caudal NEPs) and was not detected at later stages of sMN differentiation. Reduced *SOX3* expression in patient‐derived caudal NEPs was further corroborated at the protein level by quantitative proteomics. Together, these convergent findings support a model in which the CMTX3 SV disrupts the spatiotemporal regulation of *SOX3* during differentiation from iPSCs to sMNs.

Notably, complex SVs at the Xq27.1 hotspot have previously been associated with local gene dysregulation, including XX‐male sex reversal (*SOX3*) [29], X‐linked congenital generalized hypertrichosis (*FGF13*) [30], and more recently hereditary spastic paraplegia (HSP) (*SOX3*) [31], consistent with a shared regulatory mechanism at this locus. A distinct complex SV at the Xq27.1 hotspot was recently reported in an individual with clinical features overlapping CMTX3 neuropathy, accompanied by a tethered spinal cord, ophthalmological symptoms, and neuropsychiatric features [32]. Although the mechanism was not resolved, these observations raise the possibility of convergent gene dysregulation [32] and motivate testing whether *SOX3* dysregulation is also present in cells harboring this alternative genomic rearrangement.

*FGF13* was previously proposed as a high‐priority candidate gene based on its dysregulation in CMTX3 patient‐derived lymphoblasts [5]. However, in the current study, no consistent differences in *FGF13* expression were observed between patient and control cells at any stage of differentiation from iPSC to mature sMN. Among the positional candidates assessed, *SOX3* was the only gene to show reproducible CMTX3‐associated expression changes. Collectively, these findings prioritize *SOX3* as the leading candidate gene underlying CMTX3 neuropathy.

*SOX3* is the closest gene to the CMTX3 structural variant, located ~82 kb telomeric to the distal insertion breakpoint. It encodes the SOX3 transcription factor, a member of the SRY‐related HMG box (SOX) family of transcription factors that play a role in embryonic development [33, 34]. Together with SOX1 and SOX2, SOX3 forms the SOXB1 subfamily, which is highly expressed in the developing vertebrate nervous system [35–38] with a pivotal role in neural fate acquisition [27, 37, 39]. SoxB1 factors maintain neural progenitors in an undifferentiated state, thereby preventing premature terminal differentiation [40]. Beyond its well‐established role in vertebrate neurogenesis, SOX3 also contributes to gliogenesis. In the developing spinal cord, SOX3 prevents premature astrocyte differentiation [41] and is important for terminal differentiation of oligodendrocytes [42]. Interestingly, this developmental role differs from *C. elegans*, where the SoxB1 orthologue (*sox-2*) is dispensable for embryonic neurogenesis but is required for the terminal differentiation of specific cholinergic and glutamatergic neuronal subtypes [43].

Although functional redundancy among SOXB1 transcription factors has been well‐documented [40, 44, 45], *Sox3*‐null mouse models have identified developmental processes that are particularly sensitive to the loss of SOX3. These include formation of the hypothalamo‐pituitary axis, with *Sox3* knockout mice exhibiting hypopituitarism [46, 47]. SOX3 is also expressed in distinct subpopulations of undifferentiated spermatogonia, with an important role in spermatogenesis [48, 49]. Additional phenotypes in *Sox3* knockout mice include craniofacial abnormalities, CNS midline defects, and impaired fertility [46, 47, 50, 51]. Notably, marked variability in both the severity and penetrance of the *Sox3*‐null phenotypes has been reported [46, 50], and genetic background strongly modifies the phenotypic consequences of *Sox3* loss [46, 48].

In humans, pathogenic *SOX3* variants are associated with a broad phenotypic spectrum (reviewed in [52]), although *SOX3* has not been directly implicated in CMT. These variants most commonly cause variable forms of hypopituitarism, consistent with the essential role of SOX3 in hypothalamo‐pituitary development [53]. Reported pathogenic alterations include genomic duplications [53, 54] and deletions encompassing *SOX3* [55], polyalanine tract expansions [53, 56–58] and deletions [57], and missense variants [59, 60]. Clinical expressivity is highly variable, even among individuals carrying the same variant [60]. Hypopituitarism is frequently accompanied by additional features including intellectual disability [54, 56, 59], neural tube defects [54], structural brain abnormalities [53, 54, 60], and facial anomalies [56, 59], among others [59, 60]. Individuals with CMTX3 were not systematically assessed for *SOX3* loss‐of‐function phenotypes such as hypopituitarism; consequently, their absence cannot be definitively established. However, the partial, spatiotemporally restricted reduction in *SOX3* expression we report may explain the predominantly peripheral neuropathy phenotype and apparent absence of broader syndromic features associated with more severe disruption of SOX3.

Multiple duplications at the *SOX3* locus have been reported in patients with 46,XX disorders of sexual development (DSD) (SRY‐negative), including XX‐male sex reversal and XX‐ovotesticular DSD [61–69]. Importantly, genomic rearrangements which leave the *SOX3* coding sequence intact have also been associated with 46,XX DSD. This includes a deletion immediately upstream of *SOX3* [62], copy number gains occurring ~566 kb upstream of *SOX3* [70], and an interchromosomal insertion from chromosome 1, which is ~82 kb distal to this gene [29]. These variants are believed to cause altered cis‐regulatory architecture [70] and potential ectopic *SOX3* expression during gonadal development [29, 62]. Notably, the interchromosomal insertion reported by Haines et al. (2015) occurs within the quasi‐palindromic sequence at Xq27.1 [29], a recognized hotspot for pathogenic SV, including the CMTX3 interchromosomal insertion (reviewed in [7]).

Compelling support for *SOX3* as the lead candidate for CMTX3 comes from a recently reported interchromosomal insertion at the same Xq27.1 hotspot in a Danish family with HSP [31]. The insertion caused reduced *SOX3* expression in patient‐derived iPSCs, similar to our finding in CMTX3, as well as at multiple stages of forebrain neuronal differentiation [31]. Hi‐C analysis revealed regulatory rewiring at the *SOX3* locus in patient‐derived iPSCs, where the insertion insulates *SOX3* from distal enhancers [31]. Given the clinical and genetic overlap between HSP and inherited peripheral neuropathies (reviewed in [71–73]), this work provides strong independent evidence supporting *SOX3* as the most likely gene underlying CMTX3. Nevertheless, because *SOX3* has not yet been directly associated with CMT, further functional studies are required to establish whether the *SOX3* dysregulation in CMTX3 is causally responsible for the peripheral neuropathy phenotype.

*SOX3* is a key developmental transcription factor and was selectively dysregulated in early‐stage developmental cell types derived from CMTX3 patients. This temporal pattern raises the possibility that CMTX3 arises, at least partly, from disruption of peripheral nerve development, consistent with its early clinical onset [74]. Developmental mechanisms have also been implicated in other forms of CMT. For example, impaired Schwann cell differentiation has been demonstrated in both a rodent [75] and iPSC‐based [76] model of CMT1A, supporting the broader concept that disruption of developmental programmes contributes to disease pathogenesis [76]. RNA‐seq was performed on NEP to explore the broader transcriptional consequences associated with *SOX3* dysregulation in this early developmental context. In addition to confirming reduced *SOX3* expression, the analysis identified an additional 21 DEGs between CMTX3 and control NEPs. None of these genes has an established association with inherited peripheral neuropathy. Notably, three DEGs (*PLOD2*, *TMEM132C*, and *SOX3*) have mouse orthologues with evidence of nearby SOX3 binding, based on ChIP‐Seq analysis of neural progenitors [27, 77]. Although these findings identify potential downstream targets of SOX3, functional validation will be required to establish their relationship to SOX3 and relevance to CMTX3 pathogenesis.

Given ongoing debate as to whether CMTX3 is best classified as an axonal [78], intermediate [4, 5] or demyelinating [74] neuropathy, future studies should also assess candidate gene expression and regulation during Schwann cell differentiation. Although *SOX3* emerges as a strong candidate based on its spatially and temporally restricted dysregulation, the current data do not demonstrate that altering SOX3 levels drives neuronal or glial phenotypes relevant to CMTX3 (e.g., axonal outgrowth, survival, myelination, and electrophysiology). Establishing causality will require functional perturbation studies such as restoring SOX3 in patient cells, recapitulating SOX3 downregulation in control cells and developing in vivo models that reproduce the CMTX3‐like pattern of SOX3 dysregulation. In this context, an auxin‐inducible degron system could be particularly informative, providing a tunable and reversible approach to modulate SOX3 protein levels across defined developmental stages [79].

A limitation of the present study was the inability to assess the expression of miRNA genes within the CMTX3 linkage region (*MIR504*, *MIR505*, and *MIR320D2*), as the experimental design was optimized for robust quantification of coding and long noncoding transcripts. Therefore, we cannot exclude the possibility that one or more miRNAs are dysregulated by the complex insertion. A further limitation is the small number of iPSC lines analysed and the relatedness of the patient‐derived lines. All reported CMTX3 patients harboring the ~78‐kb insertion of 8q24.3 originate from three pedigrees sharing a common ancestor in which the mutation first arose [80]. As such, the observed transcriptional differences could be influenced by family‐specific background variation. Expanding to larger cohorts and/or incorporating isogenic controls would strengthen the link between CMTX3 and *SOX3* dysregulation. Although multiple differentiation timepoints were sampled, they may not capture the earliest developmental windows in which SOX3 exerts its effects on neural differentiation. Finally, bulk RNA‐seq and NanoString measure average expression across heterogeneous cell populations, which may mask subtle or subpopulation–specific changes that could be resolved using single‐cell and/or spatial transcriptomic approaches. Coupling these advanced technologies with PNS organoid models [81] of CMTX3 could further explore the impact of *SOX3* dysregulation on PNS development.

## 5. Conclusions

Overall, this study highlights the challenge of interpreting noncoding structural variants associated with inherited neuropathies. Using the first neuronal model of CMTX3, RNA‐seq and NanoString supported by quantitative proteomics converged on a reproducible, patient‐specific reduction of SOX3 restricted to early developmental stages and consistent with disrupted spatiotemporal regulation driven by the CMTX3 insertion. These findings position *SOX3* as a novel, leading candidate gene for CMTX3 and support a model in which Xq27.1 hotspot rearrangements remodel local regulatory architecture. Collectively, these data represent a significant step toward elucidating the pathomechanisms of CMTX3 and further support a model in which altered gene regulation may underlie this neuropathy.

## Funding

This work was supported by National Health and Medical Research Council Project Grant (APP11868687) awarded to M.L.K., MRFF Genomics Health Futures Mission Grant (APP2007681) awarded to M.L.K and S.V., a CMT Association Australia grant awarded to M.L.K. and A.B., and a National Health and Medical Research Council Investigator Grant (GNT2008066) awarded to J.J.D. Open access publishing facilitated by The University of Sydney, as part of the Wiley ‐ The University of Sydney agreement via the Council of Australasian University Librarians.

## Conflicts of Interest

The authors declare no conflicts of interest.

## Supporting Information

Additional supporting information can be found online in the Supporting Information section.

## Supporting information


**Supporting Information 1** Contains supporting information tables, figures, and table legends for the manuscript.

**Supporting Information 2** Contains supporting information tables relating to the RNA‐seq experiments. Table legends are contained in Supporting Information 1.

## Data Availability

The data that support the findings of this study are available on request from the corresponding author. The data are not publicly available due to privacy or ethical restrictions.
